# Factors associated with contralateral renal parenchymal volume changes after partial nephrectomy: A retrospective cohort study

**DOI:** 10.1002/bco2.70249

**Published:** 2026-07-22

**Authors:** Qilong Jiao, Jialong Song, Ben Cao, Jianwen Chen, Shaopeng Zhou, Yibo Chen, Cheng Peng, Lei Wang, Haiyi Wang, Qingbo Huang, Xu Zhang, Xin Ma

**Affiliations:** ^1^ School of Medicine Nankai University Tianjin China; ^2^ Senior Department of Urology Chinese PLA General Hospital Beijing China; ^3^ Senior Department of Nephrology, Chinese PLA General Hospital, Nephrology Institute of the Chinese PLA, National Key Laboratory of Kidney Diseases, National Clinical Research Center for Kidney Diseases Beijing Key Laboratory of Kidney Disease Research Beijing China; ^4^ Department of Radiology, First Medical Center Chinese PLA General Hospital Beijing China

**Keywords:** partial nephrectomy, prognosis, renal compensation, renal function, renal parenchyma volume

## Abstract

**Objectives:**

The objective of this study is to assess contralateral renal parenchymal volume (CRPV) changes in patients with renal cell carcinoma (RCC) after PN and identify the clinical factors associated with CRPV increase.

**Methods:**

This retrospective study included 143 RCC patients (aged 24–76 years) who underwent PN between 2017 and 2018. Preoperative and postoperative CRPV were assessed using 3D Slicer software. Patients were categorized into CRPV‐increase and CRPV‐decrease groups based on volumetric changes. Univariate and multivariable logistic regression analyses were conducted to determine independent factors influencing CRPV increase.

**Results:**

With a median follow‐up of 904 days (IQR: 605**–**1707), the overall median CRPV increase was 4.1% (IQR: −2.8% to 12.6%) relative to the preoperative volume, and 68.5% (98/143) of patients exhibited CRPV enlargement. Multivariable analysis identified postoperative acute kidney injury (AKI) as a significant independent predictor associated with CRPV increase (OR = 2.990, 95% CI: 1.050–8.513, *p* = 0.040), whereas preexisting hypertension was inversely associated with CRPV growth (OR = 0.446, 95% CI: 0.206–0.966, *p* = 0.041).

**Conclusions:**

Contralateral renal compensation following PN is relatively modest compared to radical nephrectomy. Postoperative AKI is associated with compensatory renal hypertrophy, whereas hypertension is negatively associated with this process. These hypothesis‐generating findings underscore the importance of perioperative renal function management and blood pressure control. Prospective studies are needed to validate these associations.

## 1 INTRODUCTION

Renal cell carcinoma (RCC) is one of the most common urological malignancies, accounting for approximately 2–3% of all cancers worldwide.^1^ With advancements in urological surgery, partial nephrectomy (PN) is now the standard surgical approach for localized RCC, offering oncologic efficacy comparable to radical nephrectomy (RN) while maintaining superior renal function.^1^, ^2^ The kidney possesses a remarkable ability for compensatory hypertrophy, a process where the contralateral kidney undergoes structural and functional enlargement following unilateral nephrectomy.^3^ This phenomenon, known as compensatory renal hypertrophy (CRH), primarily occurs through an increase in cell size rather than cell proliferation, contributing to enhanced overall renal function.^4^ Although the compensatory response after radical nephrectomy has been extensively studied, limited research has focused on contralateral renal compensation following PN,^5^, ^6^ and the factors influencing this process remain unclear.

Renal parenchymal volume (RPV) is a reliable indicator of kidney function and has been increasingly used to assess post‐nephrectomy renal adaptation.^7^, ^8^ Given that contralateral renal parenchymal volume (CRPV) changes after PN may reflect structural compensatory hypertrophy, which is distinct from functional compensation, investigating the determinants of CRPV variations is essential for surgical planning and postoperative management. Therefore, this study aimed to evaluate CRPV changes in RCC patients undergoing PN and identify associated clinical, surgical and pathological factors. By clarifying these determinants, we aim to gain deeper insights into renal compensatory mechanisms and provide insights into optimizing postoperative renal function preservation in PN patients.

## 2 MATERIALS AND METHODS

### 2.1 Study design and population

This retrospective study analysed patients diagnosed with RCC who underwent PN at our institution between January 2017 and December 2018. The study period (2017–2018) was selected to minimize confounding from changes in surgical techniques and CT imaging protocols. The inclusion criteria were pathologically confirmed T1‐stage RCC, availability of preoperative and postoperative contrast‐enhanced computed tomography (CT) imaging, and performance of PN using either laparoscopic partial nephrectomy (LPN) or robot‐assisted laparoscopic partial nephrectomy (RAPN). Patients with preexisting renal abnormalities (such as hydronephrosis, nephrolithiasis or large or multiple renal cysts occupying >10% of renal parenchyma), bilateral renal tumours, a solitary kidney, a history of previous nephrectomy or renal surgery, postoperative tumour recurrence or metastasis, or incomplete follow‐up imaging data were excluded from the study. Solitary simple cysts <2 cm were not excluded. All PN procedures were performed by a dedicated surgical team using a retroperitoneal approach. No patient underwent zero‐ischemia partial nephrectomy in this cohort; all had warm ischemia with clamping of the renal artery. The study was approved by the Ethics Committee of the Chinese PLA General Hospital (Internal Registration No. S2024‐011‐01). The requirement for informed consent was waived due to the retrospective nature of the study.

### 2.2 Imaging and volumetric analysis

**FIGURE 1:**
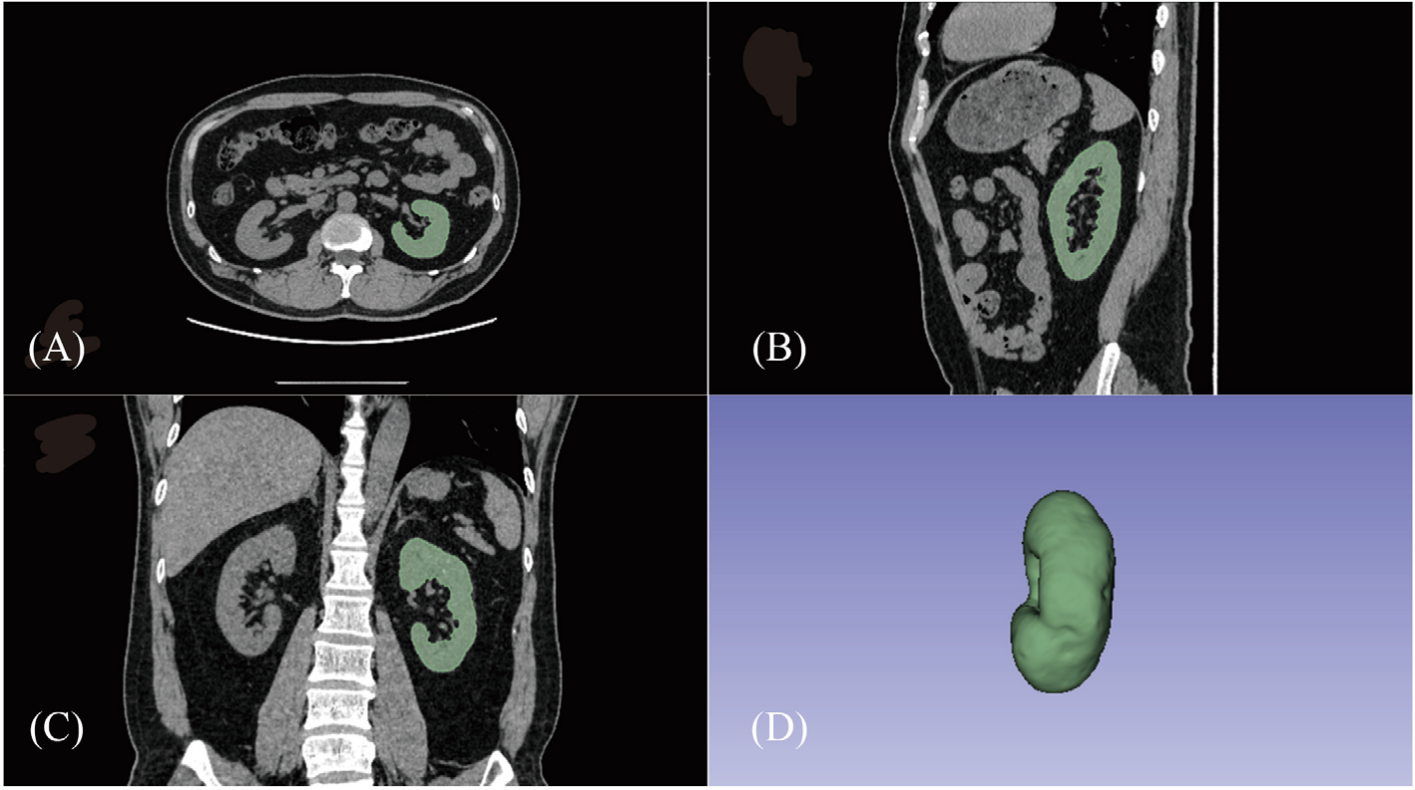
Using 3D Slicer for automatic kidney segmentation. (A)–(C) represent the transverse, sagittal and coronal planes of the CT scan, respectively; (D) is the image after three‐dimensional reconstruction.

Preoperative and postoperative renal imaging data were obtained from contrast‐enhanced CT scans performed at our institution. Postoperative CT scans were performed at a median of 12 months (IQR: 10–15 months) after surgery. 3D Slicer software Version 5.6.1 (Harvard Medical School, Boston, MA, United States) was used for volumetric segmentation of renal parenchyma (Figure 1). The region of interest (ROI) was manually defined, and an automatic flood‐filling function was employed for kidney segmentation. The bilateral renal parenchymal volume (BRPV), ipsilateral renal parenchymal volume (IRPV) and CRPV were measured before and after surgery. Preoperative IRPV was calculated by measuring the volume of the tumour‐bearing unilateral renal parenchyma and subtracting the tumour volume, which was calculated using the ellipsoid formula based on the tumour's length, width and height, as confirmed by postoperative pathological results. Tumour volume calculated by the ellipsoid formula showed a strong correlation with postoperative pathological volume (Pearson's *r* = 0.94, *p* < 0.001), confirming its validity. Other renal parenchymal volumes were directly measured using the software. All volumetric measurements were performed by one experienced radiologist to minimize measurement variability. To assess inter‐observer reproducibility, 20 randomly selected cases were re‐measured by a second experienced radiologist after a 4‐week interval. The intraclass correlation coefficient (ICC) for CRPV was 0.96 (95% CI: 0.92–0.98), indicating excellent reliability. Patients were categorized into CRPV‐increased and CRPV‐decreased groups based on postoperative volumetric changes, with changes calculated as the absolute difference relative to the preoperative baseline volume.

### 2.3 Data collection

Demographic and clinical data, including age, sex, body mass index (BMI), comorbidities (hypertension and diabetes), smoking history, preoperative and postoperative estimated glomerular filtration rate (eGFR) and postoperative pathological outcomes, were collected from electronic medical records. Hypertension was defined as a documented diagnosis in the medical record, use of antihypertensive medication or systolic blood pressure ≥140 mmHg or diastolic ≥90 mmHg on at least two separate preoperative visits. The status was assessed at baseline and remained unchanged during follow‐up unless new‐onset hypertension was recorded. Acute kidney injury (AKI) was defined according to the Kidney Disease: Improving Global Outcomes (KDIGO) criteria, as a ≥50% increase in serum creatinine within 7 days or an absolute increase of ≥0.3 mg/dl (26.5 μmol/l) within 48 h. Serum creatinine was measured daily on postoperative Days 1–3 and then as clinically indicated. Patients with preoperative eGFR < 60 ml/min/1.73m^2^ were excluded from the study. Operative data, including ischemia time, surgical duration and blood loss, were collected for analysis. The choice between RAPN and LPN was based on patient preference and availability of robotic surgical slots; no specific clinical criteria were used for allocation.

### 2.4 Statistical analysis

Continuous variables were presented as mean ± standard deviation (SD) or median (interquartile range, IQR), and categorical variables were expressed as frequencies and percentages. Comparisons between groups were performed using the independent *t* test or the Mann–Whitney *U* test for continuous variables and the *χ* ^2^ test or Fisher's exact test for categorical variables. Univariate and multivariable logistic regression analysis was conducted to identify independent predictors of CRPV increase, with results expressed as odds ratios (OR) and 95% confidence intervals (CI). Variables with *p* < 0.10 in univariate analysis were incorporated into multivariable adjustment using a stepwise forward selection method, along with clinically relevant factors. A *p* value of <0.05 was considered statistically significant. With 98 events (CRPV increase) and two variables in the final model, the events per variable were 49, exceeding the recommended minimum of 10. Statistical analyses were performed using SPSS Version 24.0 (IBM Corp, Armonk, NY, United States).

## 3 RESULTS

### 3.1 Clinical and operative data

As shown in Tables 1 and 2, a total of 143 patients met the inclusion criteria, comprising 101 males (70.6%) and 42 females (29.4%), with a mean age of 50 ± 11 years and a mean BMI of 26.0 ± 3.0 kg/m^2^. Preoperative renal function, assessed by eGFR, had a median value of 117.6 ml/min/1.73m^2^ (IQR: 105.5–136.5), which declined to 106.5 ml/min/1.73m^2^ (IQR: 92.3–121.8) at the last follow‐up, reflecting a 10.3% reduction. Regarding comorbidities, 18 patients (12.6%) had diabetes, 41 (28.7%) had hypertension and 15 (10.5%) had a history of smoking. Postoperative AKI was observed in 31 patients (21.7%). In terms of surgical approach, 85 patients (59.4%) underwent RAPN, whereas 58 (40.6%) underwent LPN. The median operative time was 126 min, and the median warm ischemia time (WIT) was 19 min. Tumour‐related characteristics included a median tumour size of 2.5 cm and a median tumour volume of 5.8 cm^3^. The majority of cases (90.2%) were classified as clear cell RCC.

**TABLE 1:** Comparison of parenchymal volume and renal function.

Variable	Overall	CRPV‐increased	CRPV‐decreased	*p*
No. of pts (%)	143 (100)	98 (68.5)	45 (31.5)	
Median IRPV, IQR, cm^3^				
Preoperative	160.7 (142.7, 188.4)	161.3 (142.7, 187.7)	160.7 (141.1, 188.9)	0.838
Postoperative	122.9 (98.7, 145.0)	122.1 (103.3, 143.7)	127.9 (94.1, 148.4)	0.927
IRPV change/%	−23.0 (−34.8, −15.7)	−22.7 (−34.3, −15.3)	−23.8 (−36.2, −16.1)	0.527
Median CRPV, IQR, cm^3^				
Preoperative	151.8 (133.6, 181.9)	151.3 (133.0, 180.4)	158.4 (134.9, 183.9)	0.468
Postoperative	161.0 (138.2, 191.9)	170.0 (146.0, 200.6)	150.2 (122.9, 172.8)	<0.001*
CRPV change/%	4.1 (−2.8, 12.6)	8.3 (3.9, 15.2)	−6.6 (−11.5, −3.3)	<0.001*
Median BRPV, IQR, cm^3^				
Preoperative	318.2 (279.1, 369.8)	316.4 (278.1, 363.5)	327.6 (281.5, 374.5)	0.503
Postoperative	280.5 (246.8, 327.6)	287.4 (257.4, 337.5)	265.9 (216.9, 321.9)	0.075
BRPV change/%	−9.3 (−16.6, −3.7)	−6.5 (−13.0, −1.63)	−16.2 (−23.6, −10.6)	<0.001*
Median eGFR, IQR, ml/min/1.73m^2^				
Preoperative	117.6 (105.5, 136.5)	117.4 (104.0, 130.6)	123.3 (108.2, 141.5)	0.179
Postoperative	106.5 (92.3, 121.8)	107.2 (89.3, 119.1)	105.7 (94.2, 127.2)	0.546
eGFR change/%	−10.3 (−18.2, −0.5)	−10.1 (−17.7, −0.3)	−10.6 (−21.0, −4.2)	0.468

Abbreviations: BRPV, bilateral renal parenchymal volume; CRPV, contralateral renal parenchymal volume; eGFR, estimated glomerular filtration rate; IRPV, ipsilateral renal parenchymal volume.

* Statistically significant difference.

**TABLE 2:** Comparison of demographic, clinicopathological and operative data.

Variable	Overall	CRPV increased	CRPV decreased	*p*
No. of pts (%)	143 (100)	98 (68.5)	45 (31.5)	
Mean age, ±SD, years	50 ± 11	49 ± 11	52 ± 11	0.107
Mean body mass index, ±SD, kg/m^2^	26.0 ± 3.0	26.2 ± 2.7	25.6 ± 3.8	0.288
Sex, male, *n* (%)	101 (70.6%)	70 (71.4%)	31 (68.9%)	0.757
Median pre‐eGFR, IQR, ml/min/1.73 m^2^	117.6 (105.5, 136.5)	117.4 (104.0, 130.6)	123.3 (108.2, 141.5)	0.179
Diabetes, *n* (%)	18 (12.6%)	12 (12.2%)	6 (13.3%)	0.855
Hypertension, *n* (%)	41 (28.7%)	23 (23.5%)	18 (40.0%)	0.042*
Postoperative AKI, *n* (%)	31 (21.7%)	26 (26.5%)	5 (11.1%)	0.038*
Smoking history, *n* (%)	15 (10.5%)	11 (11.2%)	4 (8.9%)	0.897
Surgical method, *n* (%)				0.233
Robot‐assisted	85 (59.4%)	55 (56.1%)	30 (66.7%)	
Laparoscopic	58 (40.6%)	43 (43.9%)	15 (33.3%)	
Median operative time, IQR, min	126 (100, 155)	125 (100, 155)	130 (98, 158)	0.979
Median warm ischemia time, IQR, min	19 (14, 24)	20 (14, 25)	18 (13, 23)	0.243
Median tumour size, IQR, cm	2.5 (2.0, 3.5)	2.6 (2.2, 3.5)	2.5 (1.7, 3.1)	0.070
Median tumour volume, IQR, cm^3^	5.8 (2.4, 13.7)	6.6 (2.8, 15.9)	4.6 (1.5, 9.1)	0.040*
Left‐sided tumour, *n* (%)	62 (43.4%)	40 (40.8%)	22 (48.9%)	0.366
Tumour type, ccRCC, *n* (%)	129 (90.2%)	89 (90.8%)	40 (88.9%)	0.954

Abbreviations: AKI, acute kidney injury; ccRCC, clear cell renal cell carcinoma; Pre‐eGFR, preoperative estimated glomerular filtration rate.

* Statistically significant difference.

### 3.2 Parenchymal volume and renal function changes

Based on postoperative CRPV changes, 98 (68.5%) patients were classified into the CRPV‐increased group, whereas 45 (31.5%) patients were classified into the CRPV‐decreased group. The preoperative and postoperative changes in CRPV, IRPV, BRPV and eGFR were summarized in Table 1. The CRPV‐increased group showed a significant postoperative increase with a median change of +8.3% (relative to preoperative median volume), from 151.3 cm^3^ (IQR: 133.0–180.4) preoperatively to 170.0 cm^3^ (IQR: 146.0–200.6) postoperatively. In contrast, the CRPV‐decreased group exhibited a postoperative reduction of −6.6% (relative to preoperative median volume), from 158.4 cm^3^ (IQR: 134.9–183.9) preoperatively to 150.2 cm^3^ (IQR: 122.9–172.8) postoperatively (*p* < 0.001). The IRPV decreased in both groups, with no significant difference in the percentage change between the two groups (−22.7% vs. −23.8%, *p* = 0.527). However, the BRPV decrease was significantly smaller in the CRPV‐increased group (−6.5% vs. −16.2%, *p* < 0.001). Regarding renal function, the preoperative and postoperative eGFR showed no significant difference between the two groups (*p* = 0.179 and *p* = 0.546, respectively), with a decline of approximately 10.3% after surgery.

### 3.3 Factors associated with CRPV increase

Table 2 summarized the demographic, clinicopathological and operative characteristics of the cohort. There were no significant differences in age, BMI, sex or preoperative eGFR between the two groups (all *p* > 0.05). Regarding surgical factors, the two groups had similar distributions of surgical methods (*p* = 0.233). The operative time and WIT did not significantly differ between the groups (*p* = 0.979 and *p* = 0.243, respectively). Additionally, there were no significant differences in tumour characteristics between the two groups, including tumour size, side and type (all *p* > 0.05). However, the prevalence of hypertension was significantly higher in the CRPV‐decreased group (40.0% vs. 23.5%, *p* = 0.042), whereas AKI was more commonly seen in the CRPV‐increased group (26.5% vs. 11.1%, *p* = 0.038). Furthermore, the CRPV‐increased group had a slightly larger tumour volume compared to the CRPV‐decrease group (6.6 cm^3^ vs. 4.6 cm^3^, *p* = 0.040). Although the difference in tumour volume reached statistical significance, the absolute difference was small (2.0 cm^3^), and its clinical relevance is uncertain.

In multivariable logistic regression analysis, two independent predictors of CRPV increase were identified (Table 3). Postoperative AKI was significantly associated with CRPV increase (OR = 2.990, 95% CI: 1.050–8.513, *p* = 0.040), suggesting that AKI may be linked to a compensatory hypertrophic response in the contralateral kidney. Conversely, hypertension was negatively associated with CRPV increase (OR = 0.446, 95% CI: 0.206–0.966, *p* = 0.041), indicating that preexisting hypertension might impair the compensatory response of the contralateral renal parenchyma. However, tumour volume lost significance when adjusted for other factors and was not included in the final multivariable model due to stepwise forward selection.

**TABLE 3:** Risk factors for postoperative increase of CRPV.

Variable	Univariable		Multivariable	
	OR (95%CI)	*p*	OR (95%CI)	*p*
Sex				
Female	Ref.		—	
Male	1.129 (0.524–2.434)	0.757	—	—
Postoperative AKI				
No	Ref.		Ref.	
Yes	2.889 (1.029–8.110)	0.044*	2.990 (1.050–8.513)	0.040*
Hypertension				
No	Ref.		Ref.	
Yes	0.460 (0.216–0.981)	0.045*	0.446 (0.206–0.966)	0.041*
Age, year	0.974 (0.942–1.006)	0.109	——	——
BMI, kg/m^2^	1.068 (0.946–1.207)	0.287	——	——
Tumour volume, cm^3^	1.030 (0.994–1.067)	0.104	——	——

Abbreviation: AKI, acute kidney injury.

* Statistically significant difference.

### 3.4 Supplementary analysis of AKI predictors

Table S1 presents baseline patient characteristics stratified by AKI status. In univariate analysis, WIT ≥ 25 min (*p* = 0.002) and laparoscopic surgery (compared with robot‐assisted surgery, *p* = 0.027) were associated with postoperative AKI. In multivariable analysis using stepwise forward selection, only WIT ≥ 25 min remained an independent risk factor for AKI (Table S2).

## 4 DISCUSSION

Compensatory renal hypertrophy is a well‐documented response following unilateral nephrectomy, beginning within hours and continuing until the remaining kidney reaches approximately 80% of normal bilateral renal function.^9^ In humans, because nephron formation ceases after 36 weeks of gestation, the response involves functional compensation (increased glomerular filtration rate and intraglomerular pressure) along with structural hypertrophy primarily driven by increased cell size.^4^, ^10^, ^11^

Early studies, such as that by Prassopoulos et al.,^12^ defined contralateral renal compensation based on an increase in renal parenchymal cross‐sectional area on CT scans. More recent studies have adopted CRPV as a more precise measure of contralateral renal hypertrophy.^5^, ^13^ However, there is no standardized threshold for defining ‘true’ compensatory hypertrophy, and most studies consider any increase in CRPV as indicative of contralateral renal adaptation. In our study, 68.5% exhibited CRPV increase postoperatively, suggesting that contralateral renal hypertrophy occurs in the majority of patients following PN. However, the loss of IRPV did not correlate with the presence or absence of contralateral compensation in our cohort. This finding suggests that contralateral hypertrophy is a complex biological process influenced by multiple factors, such as surgical stress, electrocautery‐induced thermal injury and ischemia–reperfusion damage. Additionally, our results indicate that even minor nephron loss can trigger contralateral compensation, implying that the relationship between renal injury and compensation may not follow a simple dose–response curve. The contralateral hypertrophy may be initiated as soon as any renal injury occurs.

Contralateral renal compensation has been reported following both PN and RN. Previous studies with 1–2 years of median follow‐up reported a median CRPV increase of 5.8–12.9% after PN, whereas the median increase after RN was significantly higher, ranging from 12.4% to 21.2%.^5^, ^6^, ^14^ In our study, the overall median CRPV increase was only 4.1%, and even among the CRPV‐increased group, the median gain did not exceed 10% (8.3%). This suggests that contralateral renal compensation following PN is relatively limited compared to RN. Further analysis revealed that patients in the CRPV‐increased group had lower preoperative eGFR and smaller preoperative BRPV than those in the CRPV‐decreased group. Interestingly, despite differences in CRPV changes, postoperative eGFR remained similar between the two groups, with a median eGFR decline of approximately 10% in both groups. These findings align with previous research, indicating that PN preserves approximately 90% of renal function.^15^ Given that renal function remained stable despite differences in CRPV changes, our results suggest that a regulatory mechanism may maintain total renal function within a stable range, with contralateral compensation playing a key role in this phenomenon. Previous studies have proposed that contralateral compensation may begin even before surgery, and multiple factors, including tumour burden and underlying comorbidities, may influence both preoperative and postoperative renal adaptation.^12^, ^16^

Our study found that hypertension negatively impacted CRPV increase following PN (OR = 0.446, *p* = 0.041). Isharwal et al.^17^ found that hypertension is associated with long‐term renal functional decline after PN, whereas Takagi et al.^5^ reported that the absence of comorbidities was significantly associated with contralateral hypertrophy. Our finding aligns with that report.^17^ Studies have shown that hypertension primarily affects the kidneys by altering renal hemodynamics and increasing glomerular pressure. Chronic hypertension can lead to glomerulosclerosis and renal parenchymal atrophy, potentially impacting both kidneys.^18^ Additionally, research has demonstrated that hypertension is a neurogenic disease driven by increased sympathetic nervous system activity,^19^ where heightened cardiac sympathetic drive can induce hypertensive cardiac hypertrophy.^20^ Similarly, renal sympathetic activation may play a role in regulating renal blood flow and compensatory hypertrophy. Tromp et al.^21^ investigated a renal artery stenosis‐induced hypertension model in sheep and found that renal sympathetic nerve activity was reduced in the contralateral kidney. This finding raises the hypothesis that renal sympathetic nerve activity might be involved, but this was not directly measured in our study. Future studies are needed to test this mechanism.

Our study identified postoperative AKI as an independent predictor associated with CRPV increase (OR = 2.990, *p* = 0.040). Previous research has shown that PN‐associated AKI is primarily transient, with minimal long‐term functional impact.^22^, ^23^ Zhang et al.^24^ found that although AKI is associated with poorer short‐term recovery, even patients with moderate‐to‐severe AKI (KDIGO Stages 2–3) demonstrated an 88–90% recovery rate. The relationship between postoperative AKI and CRPV changes remains largely unexplored, but our findings suggest that AKI is associated with subsequent contralateral hypertrophy, suggesting a potential link rather than a proven causal trigger. Because contralateral compensation can reduce the functional loss associated with AKI, this mechanism may explain why PN‐associated AKI does not significantly impair long‐term renal function. The mechanism linking AKI to contralateral hypertrophy remains speculative. One possibility is that AKI triggers the release of humoral factors such as hepatocyte growth factor or activation of the renin‐angiotensin system, which in turn stimulate contralateral renal growth. However, prolonged AKI may lead to irreversible renal damage, as shown by Bravi et al.,^25^ who demonstrated that persistent AKI increased the risk of progressive renal dysfunction following PN. Similarly, Xiong et al.^26^ reported that patients with more severe AKI exhibited greater ipsilateral renal volume loss over time. Given that contralateral compensation has an upper physiological limit, extensive nephron loss due to severe AKI may eventually exceed this adaptive threshold, leading to long‐term renal impairment.^27^

This study focused on contralateral renal compensation following PN by using 3D slicer software. Our results illustrated that postoperative AKI is associated with compensatory hypertrophy and hypertension is negatively associated with renal adaptation. Identifying AKI as a predictor of contralateral hypertrophy suggests that in the context of preserved baseline renal function, transient postoperative AKI might not be uniformly harmful and could even signal a robust compensatory response. Conversely, the negative impact of hypertension implies that strict blood pressure control may facilitate renal adaptation. These insights can guide perioperative risk stratification and patient counselling. Although current studies support considering an increase in renal parenchymal volume as an indicator of enhanced renal function, it remains an indirect observational parameter. The qualitative relationship between renal parenchymal volume and renal physiology has yet to be clearly established. Further research is needed to clarify the long‐term implications of contralateral renal changes and to develop targeted interventions for optimizing renal adaptation following nephron‐sparing surgery.

This study has several limitations. First, its retrospective design precludes causal inference. Second, it is a single‐centre study with potential selection bias. Third, we assessed structural compensation as reflected by CRPV but did not assess functional compensation such as measured GFR. Fourth, the median follow‐up of 904 days (~2.5 years) may not capture long‐term changes beyond 3 years. Fifth, the definition of AKI relied on serum creatinine, which can be affected by hydration and muscle mass.

## 5 CONCLUSIONS

This study demonstrates that contralateral renal compensation following PN is modest, with a median CRPV increase of 4.1%. Whereas postoperative AKI is associated with compensatory hypertrophy, preexisting hypertension is negatively associated with this adaptation, suggesting that renal compensation is influenced by factors beyond nephron loss, including hemodynamic alterations and neurohormonal regulation. These hypothesis‐generating findings highlight the need for perioperative renal protection strategies, such as minimizing ischemic injury and optimizing blood pressure control, to enhance postoperative renal adaptation. Further prospective studies are warranted to explore the long‐term functional implications of CRPV changes and to refine strategies for preserving renal function after nephron‐sparing surgery.

## AUTHOR CONTRIBUTIONS

**Qilong Jiao**: Conceptualization; data curation; formal analysis; methodology; visualization; writing—original draft. **Jialong Song**: Data curation; investigation; software; writing—original draft. **Ben Cao**: Data curation; validation. **Shaopeng Zhou**: Data curation; validation; writing—original draft. **Jianwen Chen**: Validation; writing—review and editing. **Yibo Chen**: Data curation; writing—original draft. **Cheng Peng**: Formal analysis; validation. **Lei Wang**: Data curation; validation. **Haiyi Wang**: Resources; validation. **Qingbo Huang**: Funding acquisition; supervision; writing—review and editing. **Xu Zhang**: Supervision; writing—review and editing. **Xin Ma**: Conceptualization; project administration; supervision; writing—review and editing.

## CONFLICT OF INTEREST STATEMENT

The authors declare no conflicts of interest.

## Supporting information


**Table S1**. Comparison of demographic, clinicopathological and operative data stratified by AKI status.
**Table S2.** Risk factors of AKI.

## Data Availability

The datasets used and analysed during the current study are available from the corresponding author on reasonable request.
