# Supplementary material for: Factors associated with contralateral renal parenchymal volume changes after partial nephrectomy: A retrospective cohort study
Source: BJUI Compass. 2026 Jul 22;7(7):e70249. doi: 10.1002/bco2.70249 (PMC13392493; doi:10.1002/bco2.70249)
Supplement: Supplementary file 1 — Table S1. Comparison of demographic, clinicopathological and operative data stratified by AKI status. Table S2. Risk factors of AKI. [file BCO2-7-e70249-s001.docx]

**Supplementary materials**

**Supplementary Table 1 - Comparison of demographic, clinicopathological and operative data stratified by AKI status.**

| Variable | AKI | Non-AKI | *P* |
| --- | --- | --- | --- |
| No. pts (%) | 31 (21.7) | 112 (78.3) |  |
| Mean age, ± SD, years | 52 ± 11 | 49 ± 11 | 0.316 |
| Mean body mass index, ± SD, kg/m^2^ | 26.0 ± 2.6 | 26.0 ± 3.1 | 0.914 |
| Sex, male, n (%) | 26 (83.9%) | 75 (67.0%) | 0.067 |
| Median pre-eGFR, IQR, ml/min/1.73m^2^ | 109.9 (97.6, 125.6) | 119.5 (107.7, 137.1) | 0.052 |
| Diabetes, n (%) | 6 (19.4%) | 12 (10.7%) | 0.328 |
| Hypertension, n (%) | 9 (29.0%) | 32 (28.6%) | 0.960 |
| Smoking history, n (%) | 2 (6.5%) | 13 (11.6%) | 0.619 |
| Surgical method, n (%) |  |  | 0.025* |
| Robot-assisted | 13 (41.9%) | 72 (64.3%) |  |
| Laparoscopic | 18 (58.1%) | 40 (35.7%) |  |
| Median operative time, IQR, min | 135 (106, 175) | 122 (100, 154) | 0.182 |
| Median warm ischemia time, IQR, min | 24 (18, 30) | 18 (13, 23) | 0.001* |
| Median tumor size, IQR, cm | 3.2 (2.5, 3.5) | 2.5 (2.0, 3.5) | 0.011* |
| Median tumor volume, IQR, cm^3^ | 9.2 (3.6, 19.2) | 5.2 (2.2, 12.0) | 0.015* |
| Left-sided tumor, n (%) | 14 (45.2%) | 48 (42.9%) | 0.981 |
| Tumor type, ccRCC, n (%) | 29 (93.5%) | 100 (89.3%) | 0.715 |

AKI=acute kidney injury; pre-eGFR=preoperative estimated glomerular filtration rate; ccRCC=clear cell renal cell carcinoma.

* Statistically significant difference.

**Supplementary Table 2-Risk factors of AKI.**

| Variable | Univariable | | Multivariable | |
| --- | --- | --- | --- | --- |
|  | OR (95%CI) | *P* | OR (95%CI) | *P* |
| Sex |  |  |  |  |
| Female | Ref. |  | —— |  |
| Male | 2.565 (0.911-7.221) | 0.074 | —— | —— |
| Surgical method |  |  |  |  |
| Robot-assisted | Ref. |  | —— |  |
| Laparoscopic | 2.492 (1.107-5.610) | 0.027* | —— | —— |
| WIT≥25 min |  |  |  |  |
| No | Ref. |  | Ref. |  |
| Yes | 4.031 (1.702-9.549) | 0.002* | 4.031 (1.702-9.549) | 0.002* |
| Age, yr | 1.019 (0.982-1.057) | 0.315 | —— | —— |
| Body mass index, kg/m^2^ | 1.007 (0.882-1.151) | 0.914 | —— | —— |
| Tumor size, cm | 1.333 (0.969-1.833) | 0.077 | —— | —— |
| Tumor volume, cm^3^ | 1.005 (0.983-1.027) | 0.666 | —— | —— |

AKI=acute kidney injury; WIT=warm ischemia time. *Statistically significant difference.
